# Blocking hexose entry into glycolysis activates alternative metabolic conversion of these sugars and upregulates pentose metabolism in *Aspergillus nidulans*

**DOI:** 10.1186/s12864-018-4609-x

**Published:** 2018-03-22

**Authors:** Claire Khosravi, Evy Battaglia, Roland S. Kun, Sacha Dalhuijsen, Jaap Visser, María Victoria Aguilar-Pontes, Miaomiao Zhou, Heino M. Heyman, Young-Mo Kim, Scott E. Baker, Ronald P. de Vries

**Affiliations:** 1 Fungal Physiology, Westerdijk Fungal Biodiversity Institute & Fungal Molecular Physiology, Utrecht University, Uppsalalaan 8, 3584 CT Utrecht, The Netherlands; 2 Microbiology, Utrecht University, Padualaan 8, 3584 CH Utrecht, The Netherlands; 3 Earth and Biological Sciences Directorate, Pacific Northwest National Laboratory, Richland, WA USA; 4 Fungal Genetics and Technology Consultancy, P.O. Box 396, 6700 AJ Wageningen, The Netherlands

**Keywords:** Central carbon catabolism, *Aspergillus nidulans*, Glycolysis, Pentose catabolic pathway, Plant biomass degradation

## Abstract

**Background:**

Plant biomass is the most abundant carbon source for many fungal species. In the biobased industry fungi, are used to produce lignocellulolytic enzymes to degrade agricultural waste biomass. Here we evaluated if it would be possible to create an *Aspergillus nidulans* strain that releases, but does not metabolize hexoses from plant biomass. For this purpose, metabolic mutants were generated that were impaired in glycolysis, by using hexokinase (*hxkA*) and glucokinase (*glkA*) negative strains. To prevent repression of enzyme production due to the hexose accumulation, strains were generated that combined these mutations with a deletion in *creA*, the repressor involved in regulating preferential use of different carbon catabolic pathways.

**Results:**

Phenotypic analysis revealed reduced growth for the *hxkA1 glkA4* mutant on wheat bran. However, hexoses did not accumulate during growth of the mutants on wheat bran, suggesting that glucose metabolism is re-routed towards alternative carbon catabolic pathways. The *creAΔ4* mutation in combination with preventing initial phosphorylation in glycolysis resulted in better growth than the *hxkA/glkA* mutant and an increased expression of pentose catabolic and pentose phosphate pathway genes. This indicates that the reduced ability to use hexoses as carbon sources created a shift towards the pentose fraction of wheat bran as a major carbon source to support growth.

**Conclusion:**

Blocking the direct entry of hexoses to glycolysis activates alternative metabolic conversion of these sugars in *A. nidulans* during growth on plant biomass, but also upregulates conversion of other sugars, such as pentoses.

**Electronic supplementary material:**

The online version of this article (10.1186/s12864-018-4609-x) contains supplementary material, which is available to authorized users.

## Background

Plant biomass is the main renewable material on earth and the major starting material for several industrial processes. In nature, *Aspergillus* degrades plant biomass polysaccharides to obtain monomeric sugars that can serve as a carbon source. *Aspergillus* is able to secrete enzymes that can hydrolyse polysaccharides into pentoses and hexoses [1], which can be taken up by the fungus. *Aspergillus* then uses a variety of catabolic pathways to efficiently convert all monomeric components of plant biomass. D-glucose is a hexose that can be phosphorylated to glucose-6-phosphate by either glucokinase (GlkA) [2] or hexokinase (HxkA) [3] in *Aspergillus nidulans*. Glucose-6-phosphate can either enter the pentose phosphate pathway (PPP) or be converted to fructose-6-phosphate by phosphoglucose isomerase (SwoM) [4] and enter glycolysis. D-fructose is phosphorylated by hexokinase (HxkA) to fructose-6-phosphate, which can also enter glycolysis [3, 5].

The critical step in bio-ethanol production is the release of sugars from plant material, because the enzymes used in this process are expensive to produce and purify. A possibility to optimize the process is to create a fungal strain that can hydrolyse the polymeric fraction but cannot utilize fermentable sugars such as D-glucose and D-fructose. With such a strain the plant degrading enzymes do not have to be produced and purified in a separate environment, but the fungus itself can be used to generate these sugars during growth on plant biomass. Such a ‘one-pot’ bioethanol process has been reported by combining cellulase production and ethanol production using a co-cultivation of *Acremonium cellulolyticus* and *Saccharomyces cerevisiae*. [6]. Another study in which *S. cerevisiae* was co-cultured with other fungi also revealed an increase in ethanol production and a reduction of the fermentation time and cost [7].

The genomes of most filamentous fungi contain all the necessary glycolytic genes for conversion of D-glucose and D-fructose. The aim of this study was to combine a carbon catabolite derepressed strain with a glucose and fructose non-consuming strain, redirecting metabolism towards consumption of other sugars (e.g. pentoses), and analyze the effect of these strains on a complex substrate. Previous phenotypic analysis of glucokinase and hexokinase mutants from *Aspergillus nidulans* demonstrated that at least one of them is required for normal growth on D-fructose or D-glucose. [3, 5]. It has also been shown that growth of an *A. fumigatus* Δ*hxkA* Δ*glkA* strain is reduced on D-glucose and D-fructose [8] and that the *A. nidulans hxkA1 glkA1* mutant is unable to phosphorylate D-glucose and D-fructose [5]. Phosphorylation of hexose sugars by both glucokinase and hexokinase was shown to play a role in mediating carbon catabolite repression (CCR) via CreA, but it was not essential [5].

In order to prevent hexose consumption we blocked entry to glycolysis of these sugars by using a hexokinase/glucokinase mutant. As accumulation of hexoses could activate carbon catabolite repression of genes encoding plant biomass degrading enzymes, we combined these mutations with a nul-mutation in *creA* caused by a partial deletion that was previously described [9]. The resulting mutant strains have been analysed on wheat bran with regard to growth, extra - and intracellular sugar accumulation, extracellular enzyme production, and gene expression.

## Methods

### Chemicals

All chemicals and reagents were acquired from Sigma-Aldrich unless otherwise noted.

### Strains, media and growth conditions

All *A. nidulans* strains used in this study are listed in Table 1 and were grown in minimal medium (MM) or complete medium (CM) with addition of a carbon source [10]. The nitrogen source used in our media is sodium nitrate (6 g/l). For all the analyses performed, two biological replicates were used, except for the metabolomics where three biological replicates were used. For the growth on plates, 1.5% (*w*/*v*) agar was added to the medium. For the production of spores, the strains were cultivated on plates containing CM supplemented with 1% xylose and incubated at 37 °C in the dark for 5 days. The spores were harvested in ACES buffer and counted with haemocytometer (THOMA) under 40× magnifications. Square plates of MM plus 1% polysaccharide or 3% crude carbon source were used for growth profile. When necessary, the medium was supplemented with pyridoxine (pyro, 0.2 g.L^− 1^), pantothenic acid (panto, 0.1 g.L^− 1^) and para-aminobenzoic acid (paba, 0.2 g.L^− 1^). The plates were inoculated with 2 μl spore solutions at 5x10^5^spores/ml and incubated at 37 °C during 3–4 days. For the transfer experiments, all the strains were pre-grown in 1 L Erlenmeyer flasks that contained 200 ml of CM plus 2% D-xylose. Liquid cultures were inoculated with 10^6^spores/ml and shaken at 250 rpm in a rotary shaker. After 16 h, the mycelium was harvested by filtration, washed twice with MM and transferred to 250 ml Erlenmeyer flasks containing 50 ml MM plus 1% wheat bran (*w*/*v*) and the appropriate supplements, at 37 °C. The mycelium was harvested by vacuum filtration and culture samples were taken after 2, 8 and 24 h of incubation. The mycelium samples were dried between tissue paper and frozen in liquid nitrogen. For RNA analysis only the 2 h samples were used. The supernatant was kept at − 20 °C for sugar and enzymatic analysis.

**Table 1:** *Aspergillus nidulans* strains used in this study. (In bold are the strains that were used in all experiments)

Name	CBS accession number	Genotype	References
**UUAM101.08**	**CBS 141343**	***y*** **A2**	**This study**
**UUAM104.47**	**CBS 141344**	***creAΔ4*** **,** ***pyro*** **A4**	**This study**
**UUAM102.37**	**CBS 141756**	***hxkA1 glkA4*** **,** ***yA2*** **,** ***paba*** **A1,** ***pyro*** **A4**	**This study**
**UUAM104.87**	**CBS 141447**	***creAΔ4 hxkA1 glkA4, y*** **A2,** ***pyro*** **A4**	**This study**
UUAM101.24		*y*A2*, pyro*A4	This study
UUAM102.71		*hxkA1 glkA4, y*A2*, pyro*A4	This study
NW299		*glkA4*, *y*A2, *pyro*A4	[5]
NW193		*hxkA1 glkA4, paba*A1	[5]
V148		*creAΔ4*, *panto*A1	This study

### Sexual crossing of *A. nidulans*

Spores of the *A. nidulans* strains were harvested in saline tween buffer. Sexual crosses of the strains were performed as described previously [11]. Selection of the progeny was based on the inability to grow on glucose and fructose for the hexokinase glucokinase double mutant and a halo around a colony grown on starch plus D-glucose for the *cre*A mutation. Selection of the triple mutant was based on the inability to grow on starch plus D-glucose and the ability to grow on starch plus D-xylose with the formation of a halo around the colony.

### Enzyme and reducing sugar assays

The concentration of the sugars in the media was measured by a reducing sugar assay as previously described [12] with modifications: samples were mixed with an equal volume of 3,5-dinitrosalicylic acid (DNS), incubated at 95 °C for 30 min and absorbance were measured at 540 nm using a microtiter plate reader (FLUOstar OPTIMA, BGM Labtech).

Extracellular enzyme activity was measured in a total volume of 100 μl using 0.01% *p*-nitrophenol (PNP) linked substrates, 20 μl of the culture samples and 50 μl of 50 mM sodium acetate pH 5.0. Samples were incubated in microtiter plates for 1 h at 37 °C. Reactions were stopped by addition of 100 μl 0.25 M Na_2_CO_3_. Absorbance was measured at 405 nm in a microtiter plate reader (FLUOstar OPTIMA, BMG Labtech). The extracellular enzyme activity was calculated using a standard curve ranging from 0 to 40 nmol *p*-nitrophenol per assay per volume. Absorbance measurements for all assays were performed in triplicate. Statistical significance for all enzyme and reducing sugar assays was determined by t-test and the Holm-Sidak method (alpha = 0.05) using GraphPad Prism version 7 for Mac, GraphPad Software, www.graphpad.com.

### Monosaccharide analysis

Monosaccharide analysis was performed for all strains, after 2 h, 8 h and 24 h of cultures in wheat bran. The culture supernatant was diluted 10-fold in MilliQ water prior to analysis. The monosaccharides were analyzed from peak areas in HPAEC-PAD (Dionex ICS-5000+ system; Thermo Scientific) equipped with CarboPac PA1 column (2 × 250 mm with 2 × 50 mm guard column; Thermo Scientific). The column was pre-equilibrated with 18 mM NaOH followed by a multi-step gradient: 0–20 min: 18 mM NaOH, 20–30 min: 0–40 mM NaOH and 0–400 mM sodium acetate, 30–35 min: 40–100 mM NaOH and 400 mM to 1 M sodium acetate, 35–40 min: 100 mM NaOH and 1 M to 0 M sodium acetate followed by re-equibration of 18 mM NaOH for 10 min (20 °C; flow rate: 0.30 mL/min). 2.5–200 mM D-glucose, D-fructose, L-arabinose, D-xylose, D-mannose, L-rhamnose, D-galactose, D-glucuronic acid, and D-galacturonic acid (Sigma-Aldrich) were used as standards for quantification [13].

### RNA extraction, cDNA library preparation and RNA-seq

Total RNA was extracted from mycelium ground in a Tissue Lyser (QIAGEN) using TRIzol reagent (Invitrogen) according to the instructions of the manufacturer. RNA integrity and quantity were analyzed both on a 1% agarose gel using gel electrophoresis and with an Agilent 2100 Bioanalyzer (Agilent Technologies). BGI Tech Solutions Co., Ltd. (Hong Kong) performed the cDNA library preparation and sequencing reactions. Throughout the process, Illumina library preparation, clustering, and sequencing reagents were used following the manufacturer’s recommendations (http://illumina.com).

On average 51 bp sequenced single-end reads were obtained, producing approximately 570 MB raw yields for each sample. Principal component analysis (PCA) was performed to show the reproducibility of our samples (Additional file 1: FigureS5).

### RNA-seq data analysis and functional annotation

We produced raw reads from the original image data by base calling. The adaptor sequences, highly ‘N’ containing reads (> 10% of unknown bases) and low quality reads (more than 50% bases with quality value of < 5%) were removed after data filtering. After data filtering, in average, ∼ 95% clean reads remained in each sample. Clean reads were then mapped to the genome of *A. nidulans* (AspGD) using SOAPALIGNER/SOAP2 [14]. In the alignment, no more than two mismatches were allowed. On average, 90% total mapped reads to the genome was achieved. The gene expression level was calculated by using FPKM method [15]. Genes with expression value higher than 150 were considered highly expressed (approximately top 5%) and differential expression was identified by CyberT bayesian ANOVA algorithm [16] with a cut-off value of fold change > 1.5 and *P*-value (corrected by multiple tests) < 0.05. The RNA-seq data have been submitted to Gene Expression Omnibus (GEO) [17] with accession number: GSE94775.

Orthologous genes between *Aspergillus niger* CBS 513.88 and *Aspergillus nidulans* FGSC A4 were obtained from AspGD [18] and FunCat [19] functional annotation was mapped accordingly. Expression higher than 150 RPKM that had a fold change of at least 1.5 between *creAΔ4 hxkA1 glkA4* vs *creAΔ4* and *hxkA1 glkA4* vs reference was analyzed regarding the functional annotation.

### Quantitative RT-PCR (qRT-PCR) validation

RNA samples for RNA-seq experiments were used for qRT-PCR. cDNA was prepared from total RNA (2.5 μg) using Thermoscript RT (Invitrogen) according to the instructions of the manufacturer. Primer Express 3.0 software (Applied Biosystems) was used to design the sequences of all primers for qRT-PCR analysis, which were tested to determine the optimal primer concentrations and efficiency. Combinations of the 50 nM, 300 nM and 900 nM (final concentration) per primer pair were checked and based on the dissociation curve the optimal primer concentration per primer pair was set. The primer sequences and optimal concentrations of the tested genes and the reference gene are listed in Additional file 2: Table S5. qPCR analysis was performed by using the ABI 7500 fast real-time PCR system (Applied Biosystems). 20 μl reactions consisted of 2 μl forward and reverse primers at optimal concentration, 20 ng cDNA sample, 10 μl ABI Fast SYBR Master Mix (Applied Biosystems), and water to a final volume of 20 μl. The cycling parameters were 95 °C for 20 s, followed by 40 cycles of 95 °C for 3 s and 60 °C for 30 s. A dissociation curve was generated to verify that a single product was amplified. Transcript levels were normalized against the glyceraldehyde-3-phosphate dehydrogenase gene (*gpdA*; AN8041), expression and quantified according to the formula 2 –(Ct gene X – Ct gpd) [20]. Control reactions included water only and RNA (i.e. not converted to cDNA to detect residual DNA in the sample). Two biological and three technical replicates were analyzed.

### Sample preparation for GC-MS metabolomics

The polar metabolites were extracted using 30 mg biomass from *A. nidulans* cell pellet, to which 200 μL MilliQ water and 800 μL of − 20 °C chloroform: methanol solution (2:1) was added. The cultures were vortexed for 10 s, sonicated for 10 min and then centrifuged (13,300×g, 4 °C, 5 min). After centrifugation 100 μL of the top aqueous layer was transferred to glass vials and dried in a vacuum concentrator (CentriVap Concentrator, Labconco). For the analysis of the polar metabolites in the spent media 100 μL of spent media was transferred directly to glass vial and dried in a vacuum concentrator.

### Chemical derivatization and GC-MS analysis

Polar metabolites from both the cell pellet and spent media were derivatized as described previously [21]. Briefly, 20 μL of methoxyamine hydrochloride in pyridine (30 mg/mL) was added to each sample, followed by 30 s of vortexing and 10 s of sonicating before incubating the samples with shaking (1000 rpm) at 37 °C for 90 min. Next, 80 μL of N-methyl-N-(trimethylsilyl) trifluoroacetamide (MSTFA) with 1% trimethylchlorosilane (TMCS) was added to each sample, subsequently, the samples were vortexed for 30 s, sonicated for 10 s and incubation at 37 °C with shaking (1000 rpm) for 30 min. Derivatized samples were then transferred to insert before being analyzed by GC-MS in random order. Blanks and FAMEs samples were also included in the analyses for background reference and RT calibration purposes, respectively.

The derivatized metabolites were separated using a HP-5MS column (30 m × 0.25 mm × 0.25 μm; Agilent Technologies, Inc.) and analyzed on an Agilent GC 7890A coupled with a single quadrupole MSD 5975C (Agilent Technologies) system. For the GC-MS analysis, 1 μL of the samples was injected (splitless) with the GC oven kept at 60 °C for 1 min after injection, subsequently, the temperature was increased to 325 °C by 10 °C/min, followed by a 5 min hold at 325 °C. Throughout the analysis the injection port temperature was kept constant at 250 °C for optimal analysis.

### GC-MS data processing

The analysis of the polar metabolites was done by processing the GC-MS raw data files using Metabolite Detector as stated previously [22]. Briefly, the retention indices (RI) calibration was carried out based on the analyses of a mixture of FAMEs internal standards (C8-C28). The data from all GCMS runs were chromatographically aligned after deconvolution and referenced to the FAMEs analyses. In the first instance metabolites were identified by matching experimental spectra to a PNNL augmented version of FiehnLib (PMID: 19928838) library [23], containing spectra and validated retention indices of more than 900 metabolites. Furthermore, unidentified metabolites were screened against the NIST14 GC-MS Spectral Library by comparing spectra alone (denoted with “NIST”). Subsequently, all metabolite identifications were manually validated to reduce deconvolution errors during automated data-processing and to eliminate false identifications. The curated data set of identified metabolites, unidentified features and their abundances for each sample was then further analyzed making use of MetaboAnalyst [24] for the multivariate data analysis (MVDA). Data were median normalized and log transformed followed by principal component, hierarchical cluster, and heatmap analysis to identify natural clustering within the data.

## Results

### Construction and verification of the mutants

A hexokinase/glucokinase double mutant (*hxkA1 glkA4*) and a triple mutant (*creAΔ4 hxkA1 glkA4*) of *Aspergillus nidulans* were obtained through sexual crosses (Table 1 and Additional file 3: Figure S1). A reference strain with yellow conidial color (UUAM101.08) was selected from the progeny of the first cross, a *hxkA1 glkA4* mutant (UUAM102.37) was selected from the second cross, and a *creAΔ4* (UUAM104.47) as well as the triple mutant *creAΔ4 hxkA1 glkA4* mutant (UUAM104.87) were selected from the third cross for further analysis (as described in the Experimental procedures, Additional file 3: Figure S1).

**Fig. 1:**
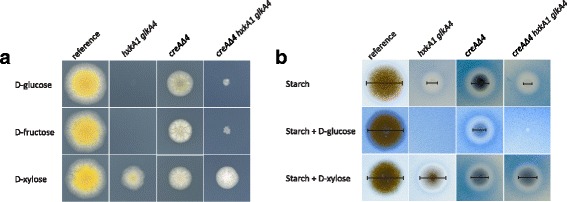
Phenotype verification of *hxkA1*/*glkA4, creAΔ4 and creAΔ4*/*hxkA1*/*glkA4.* **a** Growth of the reference and the mutants on glucose, fructose and xylose. The *A. nidulans* strains were grown on MM with 25 mM D-glucose, 25 mM D-fructose and 25 mM D-xylose for 2.5 days at 37 °C. **b** Growth of the reference and the mutants on starch. The *A. nidulans* strains were grown on MM with 1% starch with 1% starch azure, on 1% starch plus 3% D-glucose with 1% starch azure plus 3% D-glucose and on 1% starch plus 3% D-xylose) with 1% starch azure plus 3% D-xylose for 3 days at 37 °C. Spore inoculations were done with 5 × 10^5^ spores/ml. The bars represent the diameter of the colonies

Phenotypic analysis was performed to visualize the known growth defects and thus confirm the genetic mutations in our selected progeny strains. All strains were grown on D-glucose, D-fructose and D-xylose, using appropriate supplements as indicated in the experimental procedures. As expected, growth of the glucokinase/hexokinase double mutant and the triple mutant was impaired on D-glucose and D-fructose (Fig. 1a). The deletion of *creA* affected growth on all carbon sources tested (Fig. 1a and b). Moreover, its growth was reduced on starch and starch plus D-glucose compared to the reference, although a halo was observed around the colony which was smaller in the reference (Fig. 1b). Halo formation on starch plates around the colony is indicative of the activity of starch-degrading enzymes, converting non-soluble starch into soluble oligosaccharides, effectively ‘clearing’ the media. In the presence of 3% D-glucose or D-xylose, CreA represses the production of these enzymes, resulting in the absence of a halo. However, when *creA* is deleted, the halo is observed even in the presence of 3% D-glucose or D-xylose. The double and triple mutant failed to grow on starch plus D-glucose but not on starch plus D-xylose where a halo was also observed around the colony (Fig. 1b). These results confirmed the expected phenotypes of the selected strains.

### Growth profiling on plant biomass substrates revealed different phenotypes between the reference and the mutant strains

**Fig. 2:**
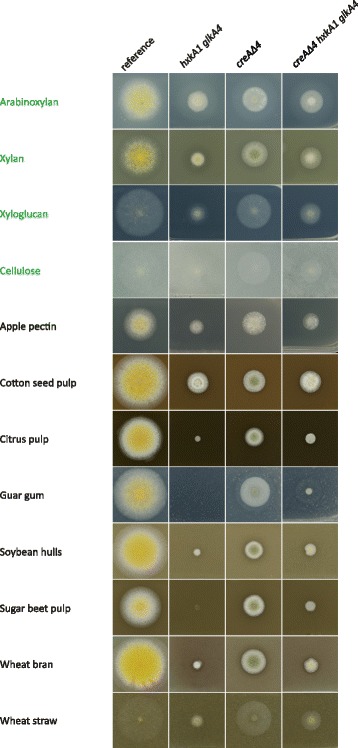
Growth of the reference*, hxkA1*/*glkA4, creAΔ4 and creAΔ4*/*hxkA1*/*glkA4* strains on a selection of pure (in green) and crude substrates. Concentrations of the substrates were 1% for apple pectin, guar gum, xylan, arabinoxylan, cellulose, xyloglucan and 3% for cotton seed, citrus pulp, wheat straw, sugar beet pulp and wheat bran

Growth of the reference strain and mutants was further analyzed on 12 pure polysaccharides and untreated agricultural waste substrates to determine the importance of glycolysis for growth (Fig. 2).

On all the selected carbon sources (except cottonseed pulp) the *creAΔ4* mutant grew more strongly than the *hxkA1 glkA4* and *creAΔ4 hxkA1 glkA4* strains (Fig. 2). The hexokinase/glucokinase negative mutant was unable to grow on guar gum and sugar beet pulp, whereas growth was strongly reduced on the other tested carbon sources. On xylan, citrus pulp, guar gum, soybean hulls and in particular wheat bran and sugar beet pulp, the carbon catabolite derepressed triple mutant grew better than the hexokinase/glucokinase double mutant (Fig. 2). Thus, growth of the double mutant on these agricultural waste substrates can be partially restored by removing carbon catabolite repression. Wheat bran is an agriculture based crude substrate and a by-product of the wheat milling industry. It consists mainly of (glucurono)arabinoxylan, cellulose, and starch, so is a good source of fermentable sugars such as D-glucose, D-xylose, and L-arabinose. Several substrates demonstrated a strong difference in growth between the double and triple mutant, but we selected wheat bran for further analysis in this study as this substrate has already been studies extensively in other studies in our lab. The composition of the polymeric and crude substrates was analyzed (Additional file 4: Table S1), but this did not reveal any obvious correlation between phenotype and the composition of the substrates. On arabinoxylan and cotton seed pulp there was no difference between the mutants. On apple pectin, *creAΔ4* showed improved growth compared to the triple mutant and the double mutant. The difference between *creAΔ4 hxkA1 glkA4* and *hxkA1 glkA4* was less visible than on wheat bran. By comparing the composition of arabinoxylan, cotton seed pulp and apple pectin to the other carbon sources tested where the differences in phenotypes were more pronounced, there was no obvious correlation between phenotype and sugar composition. This suggests that the phenotypic differences between these substrates are likely due to other factors, such as trace elements or inhibiting compounds present in the substrates.

### Sugars do not accumulate in cultures of *A. nidulans* reference and mutant strains grown on wheat bran

**Fig. 3:**
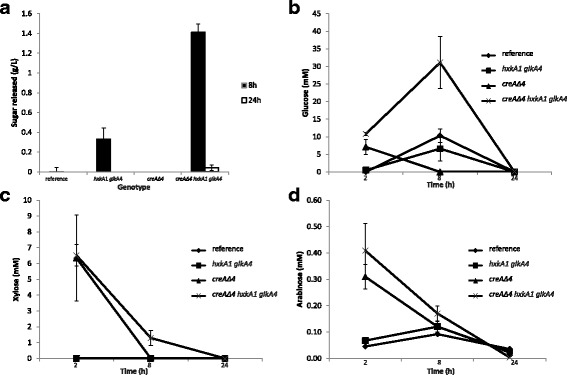
Sugar consumption by *Aspergillus nidulans* reference and mutants. All *A. nidulans* strains were transferred to 1% wheat bran. **a** Sugar levels in the extracellular medium of the reference, *hxkA1*/*glkA4, creAΔ4 and creAΔ4*/*hxkA1*/*glkA4* after 8 h and 24 h of transfer to wheat bran. The asterisk indicates a significant difference for glucose after 8 h. **b** HPAEC-PAD monosaccharide analysis of glucose, (**c**) xylose and (**d**) arabinose from *A. nidulans* reference (dark rhombus), *hxkA1*/*glkA4* (dark square), *creAΔ4* (dark triangle) and *creAΔ4*/*hxkA1*/*glkA4* (dark cross) after 2 h, 8 h and 24 h of transfer to wheat bran. A significant difference was identified between the triple mutant and *creAΔ4* for glucose after 8 h (*p* > 0.05 in students t-test). Means and SD (errors bars) were calculated from three technical replicates

Accumulation of sugars in the extracellular medium after transfer of the reference and mutants on wheat bran was analyzed based on the number of reducing groups that could be detected. The results showed that a high extracellular sugar level was present for the triple mutant after 8 h of cultivation, whereas no sugars were detected for the reference and the *creAΔ4* strain. Sugars were also present in the extracellular medium of the double mutant after 8 h of transfer to wheat bran, but at five-fold lower concentration compared to the triple mutant (Fig. 3a). After 24 h of transfer to wheat bran, the sugar concentration in the triple mutant is reduced to a very low level (Fig. 3a).

To determine any changes in sugar composition, all strains were pre-grown in liquid cultures containing MM and the necessary vitamins with xylose (since the *hxkA*/*glkA* mutant does not grow on glucose or fructose) and then transferred to the same medium with wheat bran. After 2, 8 and 24 h the monosaccharide content in the media was analyzed (Fig. 3b-d).

Significant differences in glucose concentration were found between the triple mutant and *creAΔ4* strain. After 8 h of transfer, a high level of glucose (31.1 ± 7.4 mM) was detected in the triple mutant, while in the *creAΔ4* strain glucose was not detectable (Fig. 3b). The concentration of glucose were similar in the reference and double mutant at all time points, but five-fold lower compared to the triple mutant after 8 h. In the *creAΔ4* strain*,* glucose was only detected after 2 h of transfer to wheat bran.

Xylose was not detected in cultures of the reference strain and the double mutant (Fig. 3c). However, after 2 h of transfer to wheat bran, a high level of xylose was detected in the *creAΔ4* (from 4 to 8 mM) and triple mutant (6.5 mM), which reduced in both mutants after 8 h (Fig. 3c). This trend was also observed in the *creAΔ4* and triple mutant for arabinose, but at a very low level (Fig. 3d). Low levels of arabinose were also detected in the reference and double mutant at all the time points tested (Fig. 3d). After 24 h of transfer to wheat bran no glucose, xylose or arabinose was detected in the media of any of the strains tested (Fig. 3b-d).

Thus, our analysis revealed that sugars are not progressively accumulating extracellularly but are consumed during growth of both the double and triple mutant on wheat bran. This was expected for D-xylose and L-arabinose, but the absence of free glucose after 24 h suggests alternative conversion of glucose.

**Fig. 4:**
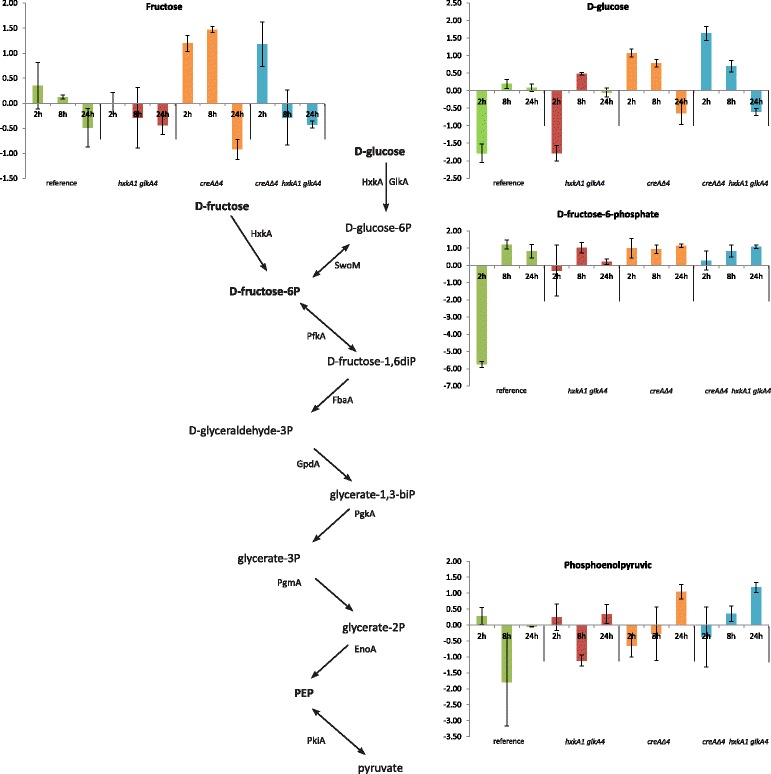
Representation of the metabolite profiles involved in the glycolysis pathway. The Y axis corresponds to the values of Additional file 5: Table S2

To trace the conversion products of glucose, GC-MS metabolomic analysis was performed on intracellular metabolite samples from the mycelia of *A. nidulans* at 2 h, 8 h and 24 h after transfer to the 1% wheat bran media. 134 metabolites were detected (Additional file 5: Table S2), of which 79 could be identified by matching them to entries in the Agilent Fiehn Metabolomics RTL and NIST GC-MS libraries. Metabolite analysis in the *creAΔ4* and triple mutants showed intracellular accumulation of glucose after 2 h. After 8 h the level is lower and after 24 h no glucose was detected (Fig. 4). For fructose, we also observed an intracellular accumulation in the *creAΔ4* and triple mutant after 2 h of transfer to wheat bran. After 8 h, the accumulation was only observed in the *creAΔ4* mutant, and at 24 h no fructose was detected. Identified metabolite profiles related to sugar catabolism in *A. nidulans* were similar for the *hxkA1 glkA4* mutant compared to the reference and for *creAΔ4* compared to the triple mutant. After 24 h of transfer to wheat bran medium, no glucose or fructose was detected in any of the strains (Additional file 5: Table S2). In summary, the hexokinase/glucokinase deficient strains when grown on a complex substrate like wheat bran did not show any indications of accumulation of glycolytic intermediates after 24 h of growth.

### Most of the up-regulated genes in the triple mutant compared to the *creAΔ4* strain are assigned to the metabolism class

RNA-sequencing was carried out using RNA isolated from xylose pre-grown mycelium from all four strains that were then transferred to MM with the necessary supplements and 1% wheat bran, to examine changes in gene expression profiles as a result of the hexokinase glucokinase defect in two genetic backgrounds (reference and *creAΔ4*). Thus in the next paragraphs, we compared the three mutants to the reference strain*,* and the same for the *creAΔ4* background.

To classify the function of the predicted *A. nidulans* genes, the functional catalogue (FunCat) was used [19]. Out of the total 10,460 genes predicted in the genome of *A. nidulans*, 5812 were associated with FunCat (component, function, and process) that were assigned to 19 main groups (see legend Additional file 6: Figure S2). There were 1798 genes up-regulated in the triple mutant compared to the *creAΔ4* strain, while 814 were up-regulated in the double mutant compared to the reference*.* Considering all up-regulated genes together, Metabolism was the major class with 39%, followed by Unclassified Proteins (34%), Transcription (5.5%) and Cellular Transport and Transport Mechanisms (5%).

Analysis of the genes from the Metabolism class was made in these two comparisons. There were 8 subgroups found in this FunCat class (see legend Additional file 6: Figure S2). C-compound and carbohydrate metabolism was the largest subgroup in the Metabolism class with 43% (307 genes) in the triple mutant vs the *creAΔ4* strain of the total of up-regulated genes (Additional file 6: Figure S2). In the double mutant, 50% of the up-regulated genes in the Metabolism class belong to the C-compound and carbohydrate metabolism subgroup (170 genes). Gene function of all genes assigned to the C-compound and carbohydrate metabolism subgroup was analyzed (Additional file 7: Table S3). Among the up-regulated genes assigned to the C-CM group, the *swoM* gene encoding the glucose-6-phosphate isomerase, two genes involved in the PCP (xylitol dehydrogenase: *xdhA* and L-arabitol dehydrogenase: *ladA*) and four genes involved in the PPP (D-ribulokinase: *rbtA*; D-ribulose-phosphate-3 epimerase: *rpeA*; glucose-6-phosphate-1-dehydrogenase: *gsdA* and transaldolase: *pppA*) were up-regulated in the triple mutant compared to *creAΔ4* (Additional file 7: Table S3, sheet 1)*.* In the double mutant (Additional file 7: Table S3, sheet 2), an additional gene involved in the PPP (6-phosphogluconate dehydrogenase: *gndA*) was up-regulated compared to the reference.

### The reduced ability to grow on wheat bran is not due to a reduced expression of CAZy genes involved in plant biomass degradation

The CAZy genes involved in plant biomass from the Glycoside Hydrolase (GH), Auxilary Activities (AAs), Carbohydrate Esterases (CEs) and Polysaccharide Lyases (PLs) families represented 1.7% of the total number of genes identified in our RNA-sequencing.

CAZy genes with RPKM values above 150 in any of the four conditions are represented in red in Additional file 8: Table S4 (sheet 2). Most of these highly expressed CAZy genes are involved in xylan and cellulose degradation. Among the significantly differentially expressed genes (with > 1.5 fold change and *p*-value < 0.05), there are only 17 down-regulated and 10 up-regulated genes in the *hxkA1 glkA4* mutant compared to the reference after a transfer to wheat bran for 2 h (Additional file 8: Table S4, sheet 1). Interestingly, 6 out of 17 genes involved in starch degradation were down-regulated in the double mutant and these genes include four α-glucosidases (*agdA*, *agdB*, *agdE* and *agdG*), one α-amylase (*amyA*) and one glucoamylase (*glaA*). In addition, two endo-xylanases (*xlnA* and *xlnC*), two endo-polygalacturonases (*pgaB* and *pgxA*) and one endo-arabinanase (*abnC*) were down-regulated. Three genes, two α-amylase genes (*amyD* and *amyG*) and one α-glucosidase gene (*agdF*) involved in starch degradation are up-regulated in the *hxkA1 glkA4* mutant as well as seven other CAZy genes that all encode exo-acting enzymes involved in degradation of xylan, cellulose/xyloglucan, galactomannan and pectin.

Overall the reference and the double mutant showed the same CAZy expression profile, which differed from the two *cre*A background strains*.* As expected, many CAZy genes are up-regulated at high transcript levels in the *creAΔ4* and the triple mutant (Additional file 8: Table S4, sheet 1). Between these two strains there are 16 genes down-regulated and 33 genes up-regulated. Most of the down-regulated genes are involved in starch (eight genes) and cellulose/xyloglucan (seven genes) degradation. We would like to note that even though expression of these genes was significantly reduced, transcript levels of many genes remain at a high level in the triple mutant. Transcript levels of seven out of 16 genes involved in arabinoxylan degradation were even more increased in the triple mutant, including the endo-xylanase gene *xlnA*, four putative β-xylosidases (AN1870, AN7864; *bxlD*, AN8477 and AN2664) and two α-arabinofuranosidases (*abfB* and An7781). In summary, these results indicate that the *creAΔ4* mutation has a stronger effect on CAZy gene expression than the metabolic mutations.

### The deletion of *creA* combined with blocking glycolysis results in an increased expression of pentose catabolic and pentose phosphate pathway genes (PCP and PPP)

**Fig. 5:**
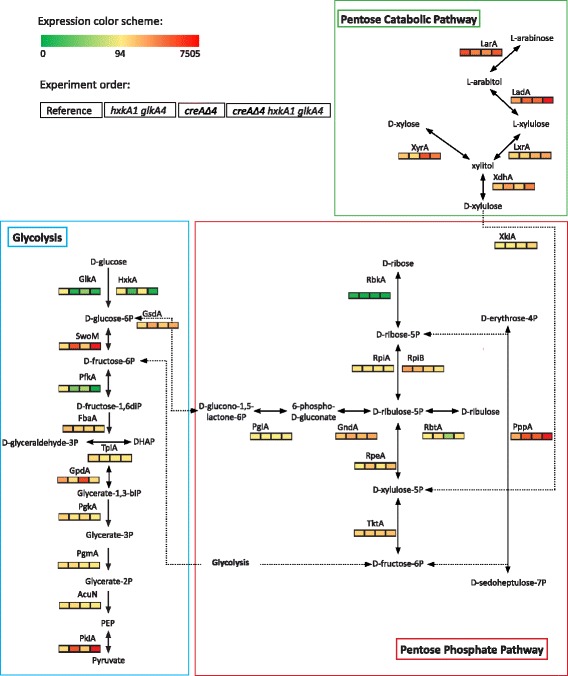
Schematic representation of the expression of genes of glycolysis, pentose phosphate pathway (PPP) and pentose catabolic pathway (PCP) in *Aspergillus nidulans* after 2 h of transfer to 1% (*w*/*v*) wheat bran*.* Gene expression values are presented under the genes and indicated by a color gradient. A decrease in expression is indicated by green squares and increased expression is indicated by a red square. Gene expressions are average values of two biological replicates. The units used is fragments per kilobase of exon per million reads mapped (FPKM). Genes involved in the glycolysis: glucokinase (*glkA*; AN8689), hexokinase (*hxkA*; AN7459), glucose-6-phosphate isomerase (*swoM*; AN6037), phosphofructokinase (*pfkA*; AN3223), fructose-biphosphate aldolase (*fbaA*; AN2875), dihydroxyacetone phosphate (DHAP), triose-phosphate isomerase (*tpiA*; AN6900), glyceraldeyde-3-phosphate dehydrogenase (*gpdA*; AN8041), phosphoglycerate kinase (*pgkA*; AN1246), phosphoglycerate mutase (*pgmA*; AN3059), enolase (*acuN*; AN5746) and pyruvate kinase (*pkiA*; AN5210). Genes involved in the PCP: L- arabinose reductase (*larA*; AN7193), L-arabitol dehydrogenase (*ladA*; AN0942), L-xylo-3-hexulose reductase (*lxrA*; AN10169), D-xylose reductase (*xyrA*; AN0423), xylitol dehydrogenase (*xdhA*; AN9064) and D-xylulose kinase (*xkiA*; AN8790). Genes involved in the PPP: glucose-6-phosphate-1-dehydrogenase (*gsdA*; AN2981), 6-phosphogluconolactonase (*pgIA*; AN0285), 6-phosphogluconate dehydrogenase (*gndA*; AN3954), D-ribulose-phosphate-3 epimerase (*rpeA*; AN7588), transketolase (*tktA*; AN0688), D-ribulokinase (*rbtA*; AN6985), transaldolase (*pppA*; AN0240), ribose 5-phosphate isomerase (*rpiA*; AN2440), ribose 5-phosphate isomerase (*rpiB*; AN5907) and ribokinase (*rbkA*; AN7995)

Most glycolytic genes showed a similar profile with a significant difference in expression between the reference and the double mutant and between *creAΔ4* and the triple mutant*.* As expected, in both mutants in which glycolysis is blocked the expression of 6-phosphofructokinase (*pfkA*), fructose-biphosphate aldolase (*fbaA*), glyceraldeyde-3-phosphate dehydrogenase (*gpdA*), phosphoglycerate kinase (*pgkA*), phosphoglycerate mutase (*pgmA*) and enolase (*acuN*) were down-regulated (lower than 0.7-fold). Exceptions were glucose-6-phosphate isomerase (*swoM*) and pyruvate kinase (*pkiA*), which were higher expressed in the hexokinase/glucokinase double mutant in both the reference and the *creAΔ4* background (Fig. 5 and Additional file 2: Table S5)*.*

Two genes from the pentose catabolic pathway (PCP), *xdhA* and *ladA*, were up-regulated in the double mutant compared to the reference and in the triple mutant compared to *creAΔ4* (> 1.5 fold, *p*-value < 0.05) (Additional file 2: Table S5). In addition, five pentose phosphate pathway (PPP) genes had a significantly higher expression in the double mutant compared to the reference and in the triple mutant compared to *creA∆4*. These encode transaldolase (*pppA*), D-ribulokinase (*rbtA*), D-ribulose-phosphate-3 epimerase (*rpeA*), 6-phosphogluconate dehydrogenase (*gndA*) and glucose-6-phosphate-1-dehydrogenase (*gsdA*). Wheat bran contains also other monosaccharides such as galactose, mannose and glucuronic acid. However, genes involved in the Leloir pathway, alternative D-galactose pathway, D- mannose pathway and TCA cycle did not show a clear effect of blocking glycolysis at the level of hexose phosphorylation.

No difference in gene expression of other genes that might convert glucose, such as glucose oxidase, glucose dehydrogenase, gluconate dehydratase and several hexokinase-like proteins (HxkB, HxkC, HxkD), was observed in the double and triple mutant compared to their reference strains (Additional file 2: Table S5) [3, 5, 25–27]. In our metabolomic data, we could not find any specific intermediates enabling us to pinpoint the alternative pathway for the conversion of glucose.

### Validation of the RNA-seq expression profiles by quantitative RT-PCR (qRT-PCR)

To validate the expression profiles obtained by RNA-seq analysis, two genes involved in the PCP (xylitol dehydrogenase, L-arabitol dehydrogenase) and one CAZy gene (α-arabinofuranosidase; *abfB*) were tested by quantitative RT-PCR (Additional file 9: Figure S3). Expression trends of the selected genes obtained by qRT-PCR confirmed those obtained with RNA-seq. The primers used in this experiment are indicated in Additional file 10: Table S6.

### Higher α-arabinofuranosidase and β-xylosidase activities were observed in the triple mutant compared to the *creA* mutant alone

The same cultures used for RNA-sequencing were also used to measure α-arabinofuranosidase, β-glucosidase, β-xylosidase and cellobiohydrolase activities in the supernatants of the cultures 8 h and 24 h after transfer to wheat bran medium. After 8 h of transfer, cellobiohydrolase (CBH) and β-glucosidase (BGL) activities were significantly higher in the reference and the *creAΔ4* mutant compared to the two other mutant strains, respectively (Additional file 11: Figure S4)*.* After 24 h of transfer to wheat bran, this difference was not observed anymore and cellulolytic activity levels were similar in all strains.

A significant increase in β-xylosidase (BXL) and α-arabinofuranosidase (ABF) activities was observed in the triple mutant in comparison with the *creAΔ4* after 8 and 24 h (Additional file 11: Figure S4)*.* No differences in BXL activity were observed between the reference and the double mutant at both time-points.

## Discussion

In this study, our aim was to create an *Aspergillus* strain that more rapidly releases hexoses from plant biomass but does not metabolize them through glycolysis, so they can be fermented by yeast more efficiently to produce bio-fuels. As a first step to achieve this, mutants for hexokinase, glucokinase, *cre*A and combinations thereof were constructed in *A. nidulans* and the resulting strains were evaluated for their ability to accumulate hexoses.

HxkA and GlkA represent the only active hexose kinases of *A. nidulans*, even though other genes encoding putative hexose phosphorylating activities, such as the putative HxkB, are present in the genome of *A. nidulans*. However, a previous study in *Aspergillus fumigatus* reported that recombinant HxkB has no sugar-phosphorylating activity [8].

The phenotype of the *hxkA1 glkA4* strains [5] was confirmed and the double mutant was unable to grow on glucose and fructose. The *creAΔ4* mutant has been described as a strongly derepressed *creA* mutant [28] and was shown to have elevated polysaccharide degrading enzyme activities [29]. Our RNA-seq data indicate that this is a transcriptionally down-regulated *creA* allele.

First, we evaluated the changes in the extracellular sugars in the reference and in all the mutant strains. A 3,5-dinitrosalicylic acid (DNS) sugar assay showed that free sugars were only present in the culture supernatant at the early time point in the triple mutant, while after 24 h of incubation, sugar levels were close to zero in all the strains. This suggests that the balance of sugar release and sugar uptake had reached a stable state. This is in agreement with the results obtained from the HPAEC analysis. At 8 h, glucose was higher in the triple mutant compared to the *creAΔ4*, but after 24 h no glucose was detected for any of the strains. Interestingly, fructose and other monosaccharides did not accumulate in all strains after 2 h, 8 h and 24 h of transfer to wheat bran. This indicates that hexoses are still being consumed in the *hxkA1 glkA4* negative strains suggesting that alternative pathways are converting them.

In the *hxkA1 glkA4* mutant, a set of genes encoding starch degrading enzymes was down-regulated at 2 h on wheat bran compared to the reference. In the triple mutant, expression of both starch and cellulose genes were reduced compared to the *creAΔ4* strain. Most glycolytic genes were down-regulated in both double and triple mutant as well suggesting blocking glycolysis caused an initial negative feedback of extracellular D-glucose release from starch and cellulose. The β-glucosidase (BGL) and cellobiohydrolase (CBH) activities also decreased after 8 h of transfer to wheat bran in both *hxkA1 glkA4* negative strains. After 24 h of incubation, BGL and CBH enzyme activities were at a similar level as their reference strain. Together with the extracellular sugar analysis, this again suggests that during growth on wheat bran over time a reorganization of central metabolism occurs in the *hxkA1 glkA4* negative strains to regulate D-glucose release and re-direct glucose conversion.

Growth profiles of the *hxkA1/glkA4* negative strains on plant biomass and pure polysaccharides were performed to determine the importance of glycolysis for growth on these substrates. Previously it was shown that the *Aspergillus fumigatus* hexokinase/glucokinase double deletion mutant failed to grow on glucose, fructose, mannose, sorbose, glucosamine, and saccharose [8]. Our results showed that in all the carbon sources tested (except arabinoxylan, xyloglucan, and cotton seed pulp) the double mutant had reduced growth compared to the triple mutant. The improved growth of our triple mutant in comparison with the double mutant observed for most substrates is most likely a consequence of the increased transcript levels of CAZymes due to the *creA∆4* mutation.

Our extracellular sugar analysis suggests that glucose and fructose may be converted through an alternative metabolic pathway. RNA-sequencing analysis allowed us to analyze in more detail the effect of the metabolic mutations and to possibly identify the pathway through which glucose and fructose were converted. Since in this biomass the amounts of galactose and mannose are low, these pathways are not expected to substantially contribute to growth. Therefore, the PCP and PPP should be the most relevant pathways necessary for growth. We assumed that the metabolic defects introduced in the strains of this study do not affect sugar transport.

Most of the glycolytic genes were down-regulated in the *hxkA1 glkA4* strains in both the reference and *creAΔ4* background. However, glucose-6-phosphate isomerase, encoded by *swoM*, which converts glucose-6-phosphate to fructose-6-phosphate in a reversible reaction, was significantly up-regulated in the strains not able to phosphorylate glucose. The pentose phosphate pathway generates besides NADPH both fructose-6-phosphate and glyceraldehyde-3-phosphate. The strong up-regulation of *swoM* indicates the necessity to increase the flux through the PPP by generating glucose-6-phosphate from the available fructose-6-phosphate pool. This is also consistent with the up-regulation of the expression of *gsdA* and *gndA*, involved in of two of three steps of the oxidative PPP, which is more evident in the hexokinase/glucokinase negative background strains. The pentoses xylose and arabinose are the main carbon sources available for catabolism and these pathways require NADPH [30]. Xylose and arabinose represent 34% and 16% (*w*/w), respectively of the wheat bran. The *xdhA* gene, encoding xylitol dehydrogenase that converts xylitol into D-xylulose, and *ladA*, encoding L-arabitol dehydrogenase that converts L-arabitol into L-xylulose, were significantly up-regulated in the *hxkA1*/*glkA4* negative strains. Also *xkiA*, encoding D-xylulose kinase that converts D-xylulose into D-xylulose-5-phosphate, was higher in the triple mutant. An important response in the non-oxidative PPP is also observed in the *hxkA1*/*glkA4* negative backgrounds which showed an increased expression of *rpeA* encoding the epimerase responsible for converting D-xylulose-5-phosphate into D-ribulose-5-phosphate and the *pppA* gene encoding a transaldolase. The ribose-5-phosphate isomerase encoding gene *rpiA* hardly changes its response in the mutant backgrounds whereas *rpiB* expression is the highest in the reference strain. Transcriptional regulation seems not to be necessary at this level. Subsequently, in the final steps of the pathway fructose-6-phosphate and glyceraldehyde-3-phosphate are generated. Glyceraldehyde-3-phosphate is then available for the final part of the glycolytic pathway which can then enter the TCA cycle.

The pool of fructose-6-phosphate generated by the catabolism of the pentoses will, in accordance with the up-regulation observed in the oxidative part of the PPP, be partially recycled whereas it will also be used for biosynthetic purposes by converting fructose-6-phosphate to glucose-1-phosphate via a glucose-6-phosphate isomerase (SwoM) and a phosphoglucomutase (PgmB). The *pgmB* gene and those encoding the subsequent steps are not up-regulated in the mutants. However, a putative UTP-glucose-1-phosphate uridylyltransferase encoding gene, *galF*, responsible for conversion of glucose-1-phosphate into UDP-glucose, was up-regulated in the triple mutant. Also, a gene encoding a putative glycogen synthase, which converts UDP-glucose into glycogen, was up-regulated in the double and triple mutant. An excess of the fructose-6-phosphate pool may lead to fructose-6-phosphate phosphatase activity. Interestingly, a fructose-2,6-bisphosphate 2-phosphatase encoding gene (*fbpZ*) was up-regulated in the triple mutant compared to the *creAΔ4* strain. This is likely to lead to lower levels of fructose-2,6-biphosphate and as a consequence to a diminished activation of the fructose-6-phosphate kinase [31]. Fructose observed intracellularly at early time points in the *creA* deletion strain and the triple mutant may arise by fructose-6-phosphate phosphatase activity which could be the consequence of an accumulation of the fructose-6-phosphate pool. However, the presence of a glucose isomerase that could convert D-glucose into D-fructose cannot be excluded. Interestingly, *dakA* (encoding dihydroxyacetone kinase) was upregulated in strains in which *hxkA* and *glkA* were mutated, especially in the *creA*d4 background. This could indicate that this enzyme is also involved in another pathway, such as fructose-6-phosphate accumulation via xylulose-5P and DHA.

These results could explain the strong up-regulation observed in the *hxkA1*/*glkA4* strains in both the reference and *creAΔ4* background of *sdhA*, encoding sorbitol dehydrogenase that converts fructose into sorbitol. Therefore, the conversion of fructose-6-phosphate may occur via a phosphatase and subsequently SdhA. Another alternative for the fructose-6-phosphate conversion might be through mannitol-1-phosphate 5-dehydrogenase, encoded by *mdpA*, which converts fructose-6-phosphate into mannitol-1-phosphate. The *mdpA* gene is highly expressed in all strains. The accumulation of mannitol in *A. nidulans* when cultivated on glucose or fructose due to mannitol-1-phosphatase activity is well known [32–35]. Extracellular metabolite analysis showed high levels of mannitol in the *creAΔ4* and in the triple mutant after 2 h of transfer to wheat bran (data not shown).

In summary our hypothesis is that xylose and arabinose are converted into fructose-6-phosphate and glyceraldehyde-3-phosphate. Fructose-6-phosphate will be converted into glucose-6-phosphate through the PPP, into mannitol through MpdA and mannitol-1-phosphatase as well as sorbitol through a phosphatase and subsequently SdhA. Expression data suggests the latter pathway to prevail.

The RNA-seq results were also used to evaluate the expression of other metabolic genes that might convert glucose. Glucose oxidase activity would result in the formation of D-gluconate and hydrogen peroxide. Our RNA-seq data did not show a significant up-regulation in the double mutant compared to the reference strain and in the triple mutant compared to the *creA* mutant. Also, in the metabolomics analysis we did not detect gluconate. Since glucose seems not to be oxidized, another option would then be reduction to glucitol. In the metabolomic analysis we also did not detect glucitol. Glucose could also be converted into trehalose, since we observed formation of glucose-6-phosphate from the PPP whereas also glucose-1-phosphate and UDP-glucose biosynthesis is intact. But most likely, glucose is either directly reduced or converted to fructose followed by production of polyols.

## Conclusion

In conclusion, this study provides an in-depth analysis of the effect of a hexokinase/glucokinase double mutant at the gene expression level during growth of *A. nidulans* on an agricultural waste product. Although glucose and fructose initially accumulate, these hexoses are converted through alternative metabolic pathways. A more detailed metabolomic analysis is required to determine which pathways are actually used and what the final products are. The deletion of *creA* in combination with a hexokinase glucokinase deficiency results in an increased expression of the PCP and PPP. This indicates that the reduced ability to use hexoses as carbon source has resulted in a shift towards the pentose fraction of wheat bran as the major carbon source to support growth.

## Additional files

Additional file 1: **Figure S5.** Principal component analysis (PDF 165 kb)

Additional file 2: **Table S5.** Expression of known gene involved in central carbon metabolism in *Aspergillus nidulans. (XLSX 32 kb)*

Additional file 3: **Figure S1.** Scheme of sexual crossing in *Aspergillus nidulans. (PDF 185 kb)*

Additional file 4: **Table S1.** Composition of the substrates. (XLSX 13 kb)

Additional file 5: **Table S2.** Intracellular metabolism of *Aspergillus nidulans* during growth in wheat bran. (XLSX 84 kb)

Additional file 6: **Figure S2.** Functional classification of *Aspergillus nidulans* genes according to FunCat. (PDF 151 kb)

Additional file 7: **Table S3.** Functional classification of *A. nidulans* genes belonging to the C-compound and carbohydrate metabolism subclass according to FunCat. (XLSX 60 kb)

Additional file 8: **Table S4.** Expression of selected CAZymes involved in the degradation of plant biomass in *Aspergillus nidulans. (XLSX 63 kb)*

Additional file 9: **Figure S3.** Expression patterns and validation of RNA-sequencing analysis by qPCR. (PDF 325 kb)

Additional file 10: **Table S6.** Primers used in this study to generate the gene fragments for qRT-PCR analysis. (XLSX 10 kb)

Additional file 11: **Figure S4.** Comparison of extracellular enzyme activities in reference and disruption strains. (PDF 169 kb)

## Abbreviations

AA: Auxiliary activities
CAZy: Carbohydrate active enzyme database
CCR: Carbon catabolite repression
CE: Carbohydrate esterase
creA: Carabon catabolite repressor encoding gene
GH: Glycoside hydrolase
glkA: Glucokinase encoding gene
hxkA: Hexokinase encoding gene
PCP: Pentose catabolic pathway
PL: Polysaccharide lyase
PPP: Pentose phosphate pathway
