# Supplementary material for: Blocking hexose entry into glycolysis activates alternative metabolic conversion of these sugars and upregulates pentose metabolism in Aspergillus nidulans
Source: BMC Genomics. 2018 Mar 22;19:214. doi: 10.1186/s12864-018-4609-x (PMC5863803; doi:10.1186/s12864-018-4609-x)
Supplement: Supplementary file 1 — Figure S5. Principal component analysis (PDF 165 kb) [file 12864_2018_4609_MOESM1_ESM.pdf]

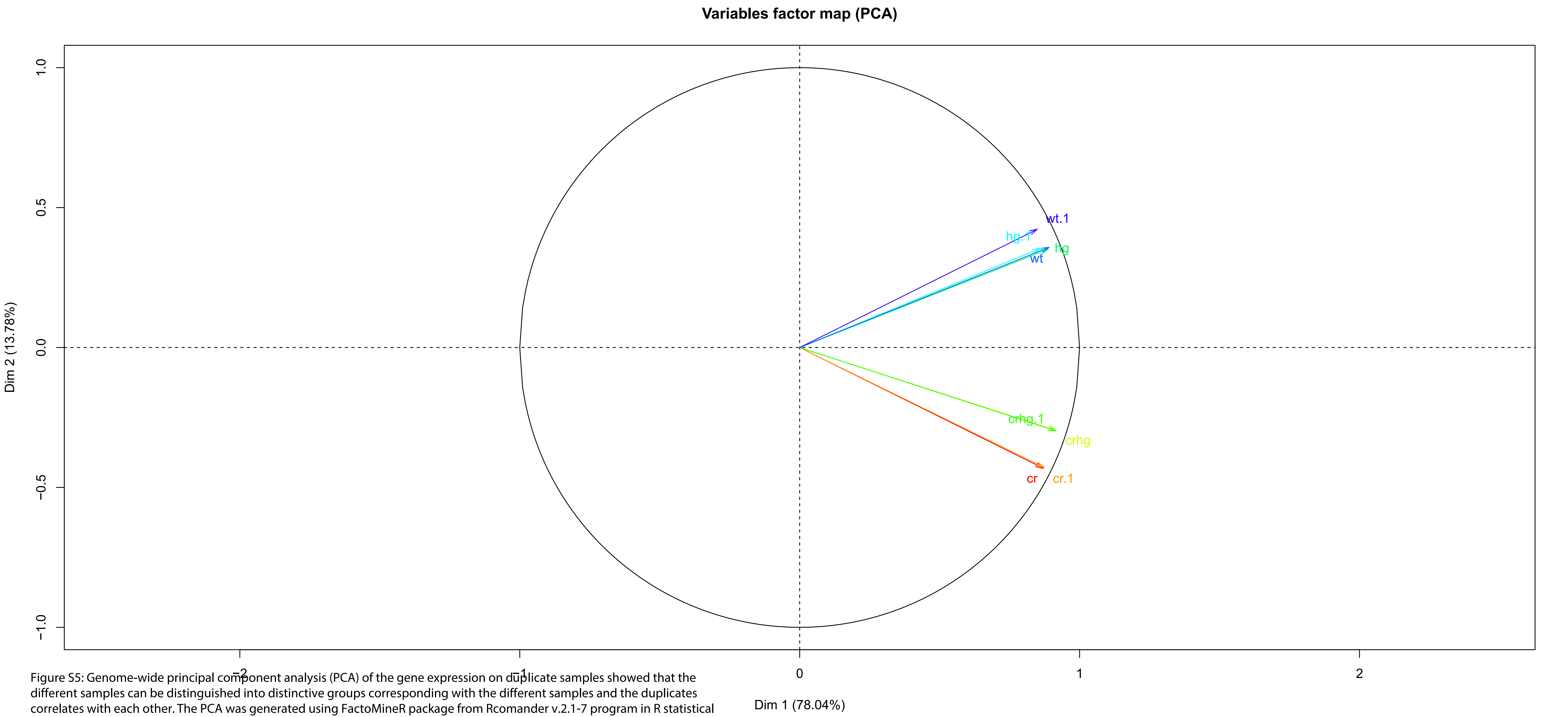

Figure S5: Genome-wide principal component analysis (PCA) of the gene expression on duplicate samples showed that the different samples can be distinguished into distinctive groups corresponding with the different samples and the duplicates correlates with each other. The PCA was generated using FactoMineR package from Rcomander v.2.1-7 program in R statistical language and envi-ronment 3.1.2.
