# Supplementary material for: Blocking hexose entry into glycolysis activates alternative metabolic conversion of these sugars and upregulates pentose metabolism in Aspergillus nidulans
Source: BMC Genomics. 2018 Mar 22;19:214. doi: 10.1186/s12864-018-4609-x (PMC5863803; doi:10.1186/s12864-018-4609-x)
Supplement: Supplementary file 3 — Figure S1. Scheme of sexual crossing in Aspergillus nidulans. (PDF 185 kb) [file 12864_2018_4609_MOESM3_ESM.pdf]

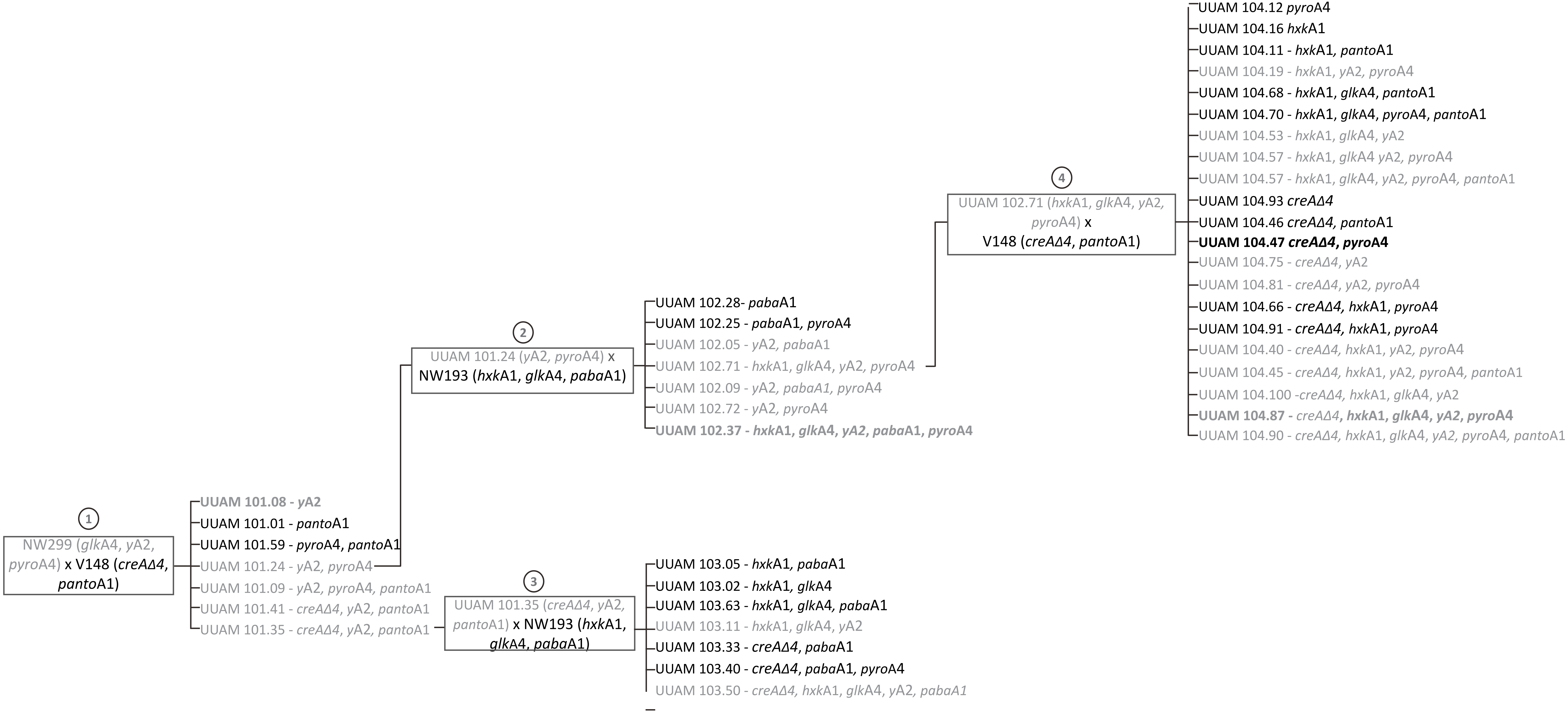

Figure S1. Schema of sexual crossing in *A. nidulans*. The strains were crossed according to schema. Strains resulting from cross 1-4 were named UU-AM101 – UU-AM104 respectively. We isolated progeny as separate colonies of the parental strains or recombinant genotypes. The strains used in this study are in bold, light grey color (*y*A2) and dark color (absence of *y*A2)
