# Supplementary material for: Blocking hexose entry into glycolysis activates alternative metabolic conversion of these sugars and upregulates pentose metabolism in Aspergillus nidulans
Source: BMC Genomics. 2018 Mar 22;19:214. doi: 10.1186/s12864-018-4609-x (PMC5863803; doi:10.1186/s12864-018-4609-x)
Supplement: Supplementary file 6 — Figure S2. Functional classification of Aspergillus nidulans genes according to FunCat. (PDF 151 kb) [file 12864_2018_4609_MOESM6_ESM.pdf]

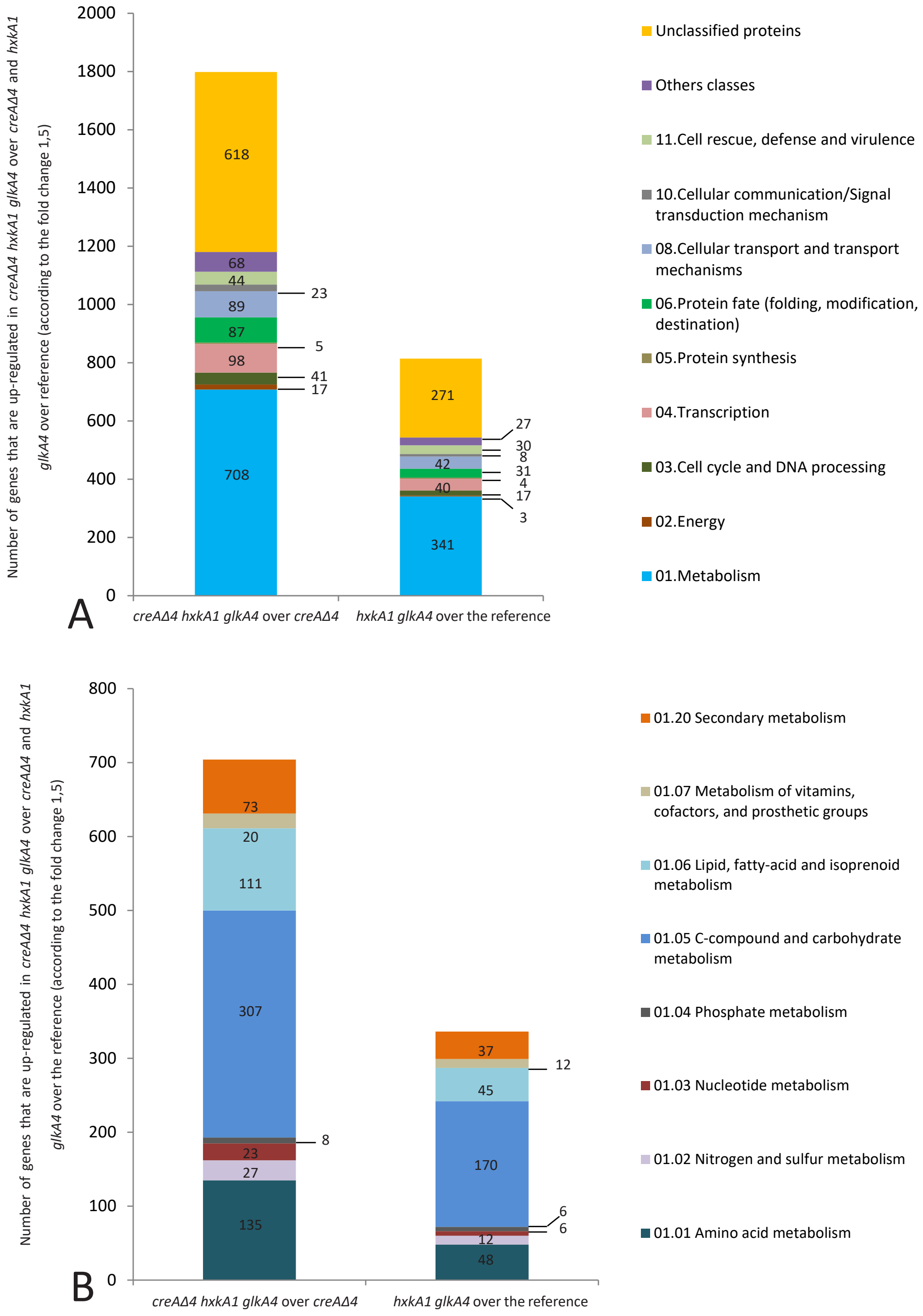

Figure S2. Functional classification of *A. nidulans* genes according to FunCat. (A) FunCat classes are, 01, Metabolism; 02, Energy; 03, Cell cycle and DNA processing; 04, Transcription; 05, Protein synthesis; 06, Protein fate; 08, Cellular transport and transport mechanisms; 10, Cellular communication/signal transduction mechanism; 11, Cell rescue, defense and virulence; Others classes: (13, Regulation of/interaction with cellular environment; 14, Cell fate; 25, Development (systemic); 29, Transposable elements, viral and plasmid proteins; 30, Control of cellular organization; 40, Subcellular localization; 45, Tissue localization; 63, Protein with binding function or cofactor requirement; 67, Transport facilitation) and Unclassified proteins. The numbers represent the number of genes in each category. The lengths of the bar are proportional to the number of genes in each category. (B) Metabolism (01): amino acid metabolism (01.01); nitrogen and sulfur metabolism (01.02); nucleotide metabolism (01.03); phosphate metabolism (01.04); C-compound and carbohydrate metabolism (01.05); lipid, fatty-acid and isoprenoid metabolism (01.06); metabolism of vitamins, cofactors, and prosthetic groups (01.07); secondary metabolism (01.20).
