# Supplementary material for: Blocking hexose entry into glycolysis activates alternative metabolic conversion of these sugars and upregulates pentose metabolism in Aspergillus nidulans
Source: BMC Genomics. 2018 Mar 22;19:214. doi: 10.1186/s12864-018-4609-x (PMC5863803; doi:10.1186/s12864-018-4609-x)
Supplement: Supplementary file 9 — Figure S3. Expression patterns and validation of RNA-sequencing analysis by qPCR. (PDF 325 kb) [file 12864_2018_4609_MOESM9_ESM.pdf]

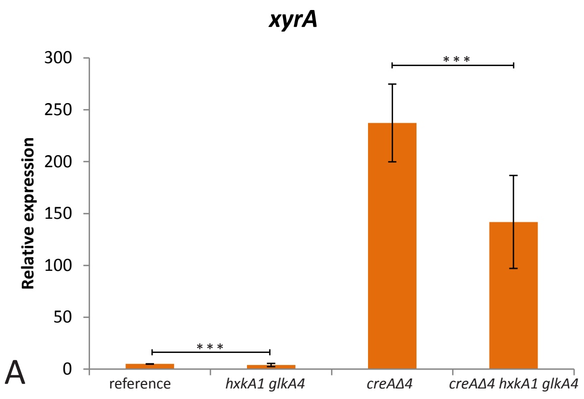

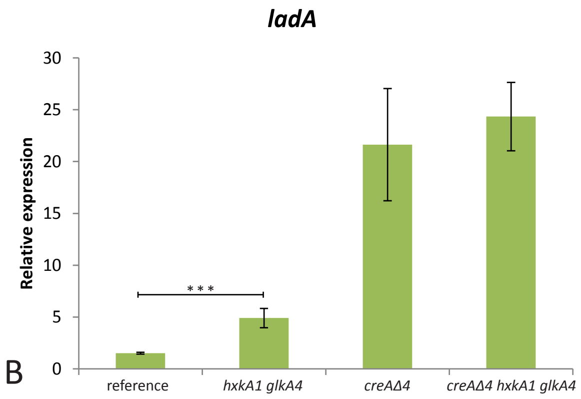

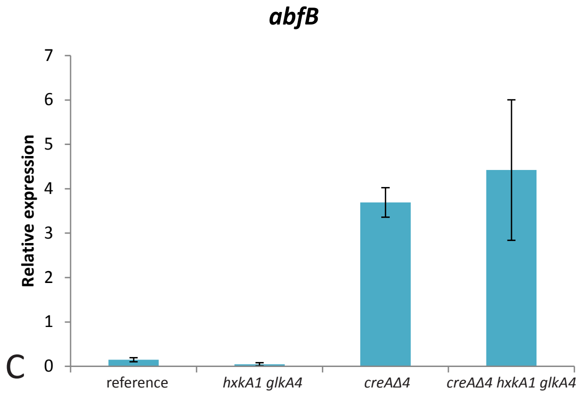

Figure S3. Expression patterns and valida ion of RNA-sequencing analysis by qPCR.
Two genes involved in the PCP: *xyrA* (A), *ladA* (B) and one gene involved in plant biomass degradation by *A. nidulans: abfB* (C). Means and SD (error bars) were calculated from two biological replicates with three technical replicates. Student t-test were performed between the reference and *hxkA1 glkA4*and between*creAΔ4* and*creAΔ4 hxkA1 glkA4* $p<0.0005$ (***).
