# Supplementary material for: Blocking hexose entry into glycolysis activates alternative metabolic conversion of these sugars and upregulates pentose metabolism in Aspergillus nidulans
Source: BMC Genomics. 2018 Mar 22;19:214. doi: 10.1186/s12864-018-4609-x (PMC5863803; doi:10.1186/s12864-018-4609-x)
Supplement: Supplementary file 11 — Figure S4. Comparison of extracellular enzyme activities in reference and disruption strains. (PDF 169 kb) [file 12864_2018_4609_MOESM11_ESM.pdf]

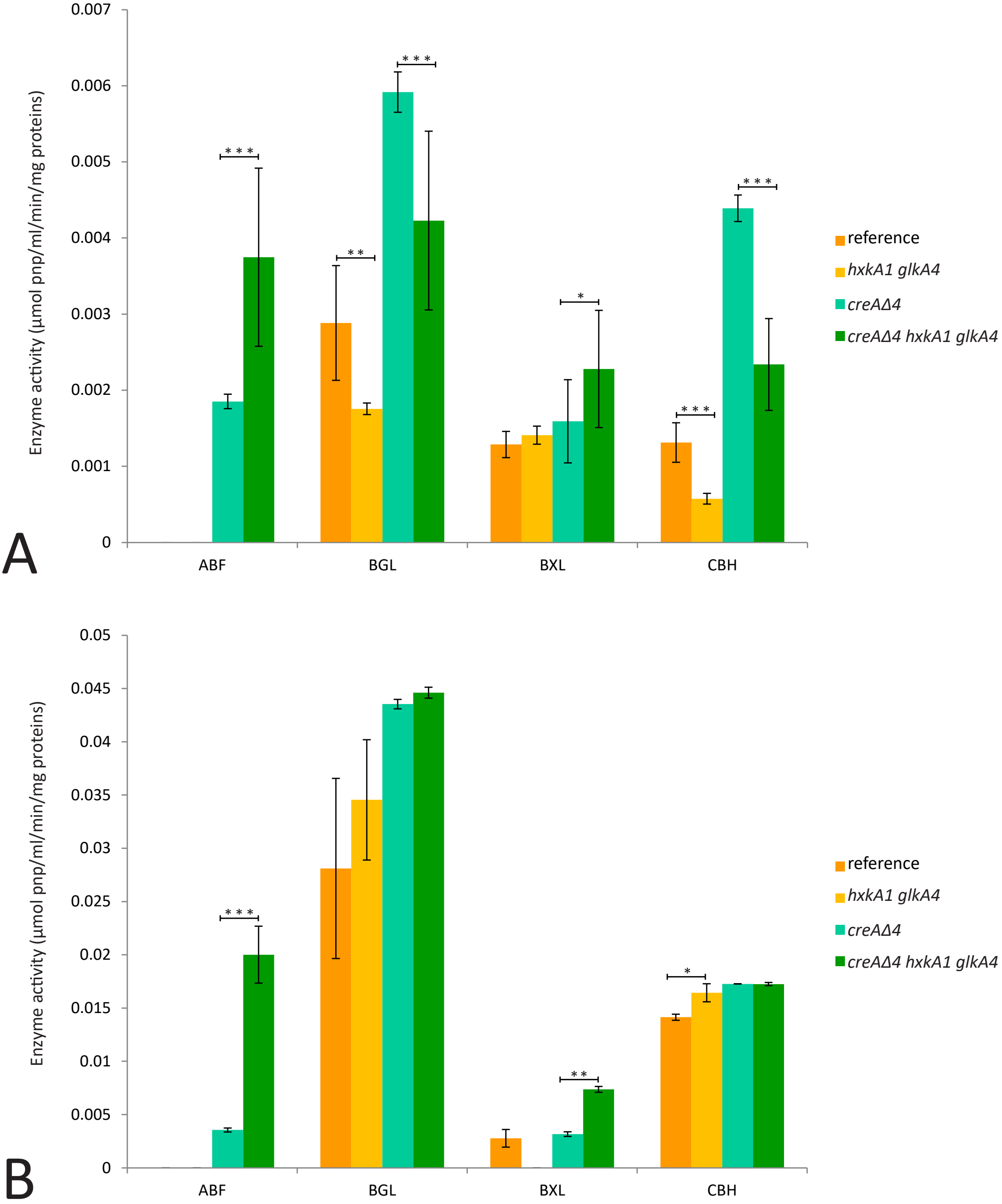

Figure S4. Comparison of extracellular enzyme activities in wild-type and disruption strains. Reference, *hxkA1 glkA4*, *creAΔ4* and *creAΔ4 hxkA1 glkA4 A. nidulans* strains were transferred for 8 hours (A) and 24 hours (B) in wheat bran. Enzyme activities of α-arabinofuranosidase (ABF), β-glucosidase (BGL), β-xylosidase (BXL) and cellobiohydrolase (CBH) in µmol pnp/ml/min/mg proteins. Means and SD (error bars) were calculated from two biological replicates with three technical replicates. Students t-test was performed between reference and the double mutant and between *creAΔ4* and the triple mutant. $p<0.005$ (***), $p<0.05$ (**), $p<0.05$ (*).
